# Left Atrial Malignant Fibrous Histiocytoma with Right Atrium Invasion

**DOI:** 10.22088/cjim.10.2.228

**Published:** 2019

**Authors:** Mehrnoush Toufan-Tabrizi, Rezayat Parvizi, Najmeh Reshadati, Behrooz Shokouhi

**Affiliations:** 1 Cardiovascular Research Center, Tabriz University of Medical Sciences, Tabriz, Iran; 2 Pathology Department, Tabriz University of Medical Sciences, Tabriz, Iran

**Keywords:** Cardiac tumors, Histiocyroma, Palpitation, Surgery

## Abstract

**Background:**

Primary cardiac tumors are rare (0.001 to 0.03%). Malignant tumors account for 25%, of which 75% are cardiac sarcomas.

**Case Persentation:**

Here, we report a case of a 57-year-old male with palpitation and history of left atrial (LA) myxoma resection presented to cardiology clinic for postsurgical follow up and transthoracic echocardiography revealed a large non-homogenous mass in LA with right atrium invasion, which was confirmed by trans-esophageal echocardiography. The patient underwent surgical resection of tumor and the pathological diagnosis was malignant fibrous histiocytoma (MFH).

**Conclusion:**

MFH could be asymptomatic and the diagnosis be established as a surgical or complementary examination. In patients with history of myxoma resection and cardiac masses, further evaluation is recommended.

Primary cardiac sarcomas are rare tumors that represent 20% of all primary cardiac tumors (1) and constitute the most common subtype (75%) of cardiac malignant tumors (2). These patients most commonly present with palpitation and dyspnea, but some are asymptomatic and diagnosed incidentally (3). Although malignant tumors are usually detectable with echocardiography, the diagnosis of sarcoma is rarely made until after surgical intervention (2). Potential complications are arrhythmia, thrombosis, pulmonary embolism and rupture of the dilated appendage (4, 5). The disease is poor prognosis with a mean survival of 9.6 to 16.5 months (1, 2, 6, 7). Although adjuvant chemotherapy and radiotherapy have been used, the standard treatment remains complete surgical resection of tumor (2). We present a case of malignant fibrous histiocytoma in a 57-year old man.

## Case presentation

**Figure 1:**
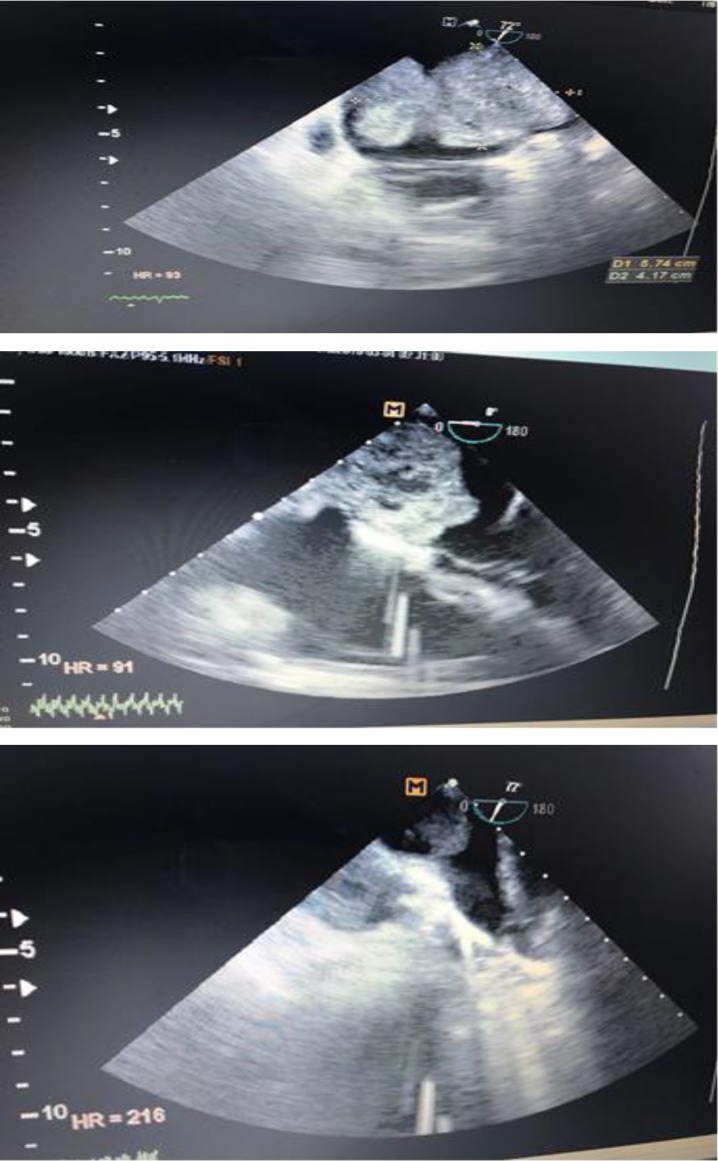
TEE midesophageal view; A: large semimobile non-homogenous mass in LA attached to IAS with size 5.74 *4.17 cm. B: large non homogenous mass in LA attached to IAS with lucent part within it. C: TEE midesophageal view in 77 ' showed: non-homogenous mass in La with extension to left pulmonary veins

A 57-year-old man with the history of left atrial (LA) myxoma presented to cardiology clinic every 6-month follow-up visit. He underwent LV myxoma resection 2 years ago. He had palpitation, but did not suffer from chest pain or dyspnea. Past medical history was positive for smoking with no history of chest trauma. On physical exam, the patient was awake; he had a regular S1 S2 with a S4 gallop, with normal breathing sounds all over the lungs. Initial EKG was normal without ischemic changes. The chest radiography showed global enlargement of the cardiac area and distributed broncovascular view. Transthoracic echocardiogram (TTE) was performed, revealing a normal right and left ventricular size and function (LVEF= 55-60%), and a large non-homogenous mass with lucent part within it suggestive of infarct part of tumor attached to interatrial septum (6×4cmm, mass area=13cm^2^) (figure 1), most probably myxoma, severe eccentric MR due to prolaptic P2 scallop, moderate TR, RVSP=70 mmHg, severe PH, and mild PI.

These findings were confirmed by trans-esophageal echocardiography (TEE). The right atrium was normal and on Doppler study, significant diastolic restriction to the filling flows of both ventricles was not found. Pulmonary CT angiography with 64 multi-slice CT scan and VRT and MIP reconstruction revealed a soft tissue within LA with 78 mm diameter with LA expansion and pressure effect on neighboring chambers. There was single lymph node in thorax inlet (31 mm). There was not pulmonary, lobar and segmental main artery feeling defect. Laboratory examinations were normal except for high level of serum pro-BNP (33964 pg/ml).

**Figure 2:**
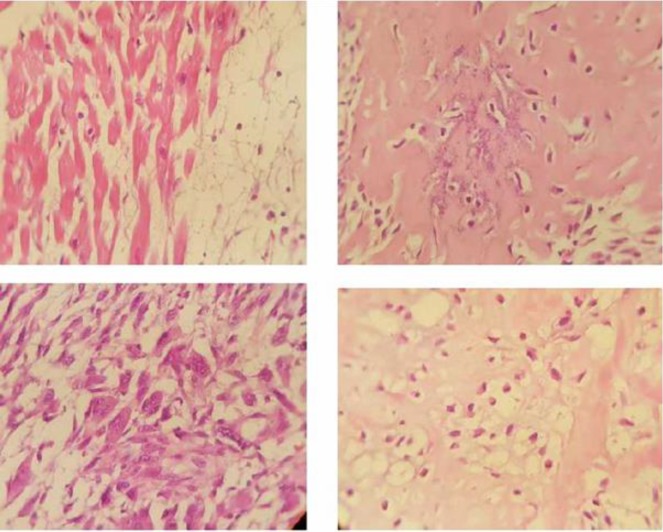
Pathologic studies: proliferation of neoplastic spindle- shaped fibroblastic cells having fascicular patterns or arranged haphazardly with scattered multinucleated giant cells. The cells have pink cytoplasms and atypical fusiform nuclei (H&E ×400)

The patient underwent surgical resection of tumor. Tumor resection was performed with the patient under cardiac arrest with cardiopulmonary bypass. Pathology results showed proliferation of neoplastic spindle- shaped fibroblastic cells having fascicular patterns or arranged haphazardly with scattered multinucleated giant cells. The cells have pink cytoplasms and atypical fusiform nuclei (figure 2); all indicative of malignant fibrous histiocytoma (MFH). After operation and moving to ICU, the patient was complicated by high grade fever, pneumonia, increasing BUN and creatinine and finally, acute renal failure. Unfortunately the patient expired 3 days after the operation with ARF and respiratory failure associated with pseudomonas pneumonia. 

## Discussion

Most of adult primary cardiac tumors are benign (75%). Moreover, 50% of RA primary tumors are malignant while a majority of LA tumors are benign and mostly consist of myxomas (50–75%) (8). 

Primary cardiac sarcomas are rare and represent 20% of all primary cardiac tumors. Symptoms depend on the chambers and the cardiac structures involved. Transthoracic echocardiography is commonly used to identify a cardiac mass. Primary MFH has been reported as the second most common primary cardiac sarcoma which usually occurs at age range of 14 to 77 years (9). 

Undifferentiated sarcomas account for one-third of all cardiac sarcomas and have been incorporated in the malignant fibrous histiocytoma/ pleomorphic sarcoma subgroup (1, 2). Cardiac MFH occur and is defined more frequently in LA (9).

 The case we report differs from those in the consulted literature in regard to anatomical features and more extended tumor size, as presented with involvement of LA and RA wall. As reported in previous literature, the patient may be asymptomatic and the diagnosis was established as a surgical or complementary examination (3).

 Our patient had only palpitation and the diagnosis was made by transthoracic echocardiography and contrast TEE led to accurate diagnosis. Elective treatment regimen of cardiac sarcoma includes complete surgical excision when possible, followed by radiotherapy and chemotherapy (1). The presented case was interesting for the presence of huge LA malignant fibrous histiocytoma with RA invasion; so, individualization of the treatment according to clinical features and complementary examinations (valuing the presence of thrombi, compression of adjacent structures, and associated diseases) was required. 

Therefore after discussing the treatment options with the patient, we decided for surgical tumor resection. As mentioned, the prognosis of cardiac sarcomas is very poor (6, 7). However, our case was complicated and expired 3 days after the operation.
